# Supplementary material for: Splice-Junction-Based Mapping of Alternative Isoforms in the Human Proteome
Source: Cell Rep. Author manuscript; Available in PMC 2020 Jan 15. (PMC6961840; doi:10.1016/j.celrep.2019.11.026)
Supplement: 2 [file NIHMS1546469-supplement-2.zip › DF1/PXD010154/Testis/Testis_12_KIAA1210_SLTATQVEPKEEEPNLPLVSEEEK.pdf]

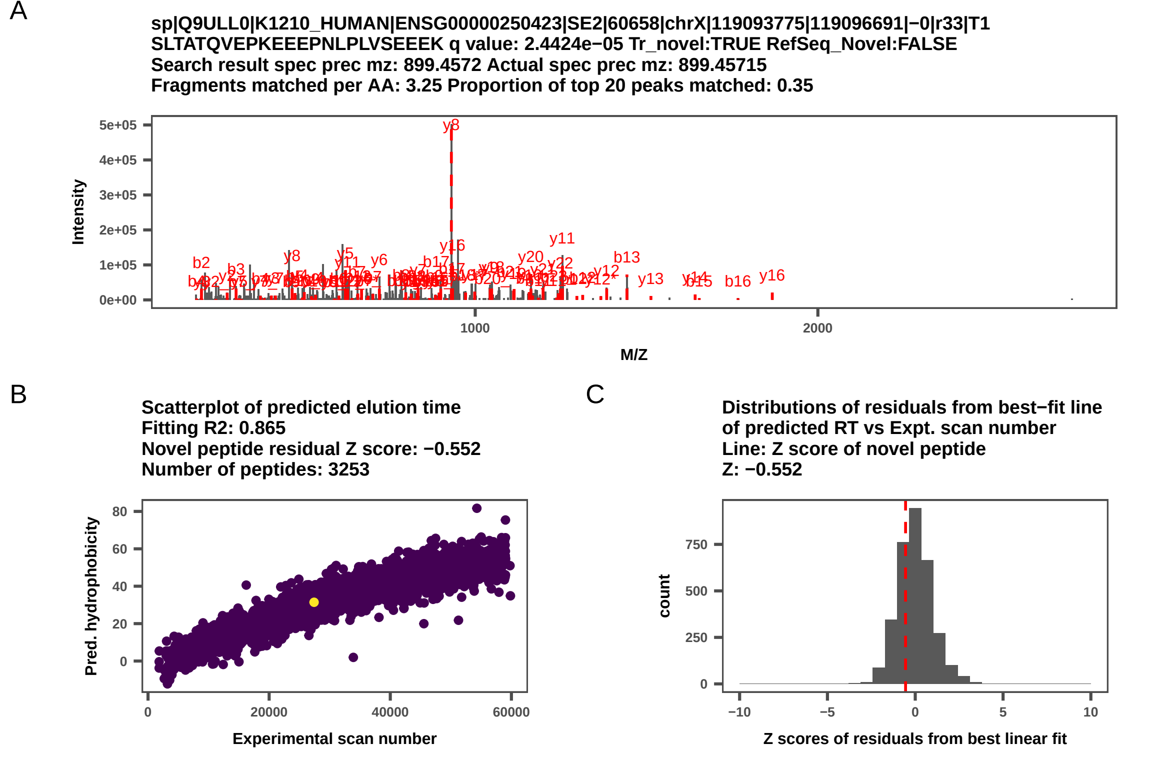
A
sp|Q9ULL0|K1210_HUMAN|ENSG00000250423|SE2|60658|chrX|119093775|119096691|−0|r33|T1
SLTATQVEPKEEEPNLPLVSEEEK q value: 2.4424e−05 Tr_novel:TRUE RefSeq_Novel:FALSE
Search result spec prec mz: 899.4572 Actual spec prec mz: 899.45715
Fragments matched per AA: 3.25 Proportion of top 20 peaks matched: 0.35
Intensity
5e+05
4e+05
3e+05
2e+05
1e+05
0e+00
y8
y16
y11
y20
y22
b13
y12
y13
y14
b15
b16
y16
b2
b3
y8
y5
y11
y6
b17
M/Z
1000
2000
B
Scatterplot of predicted elution time
Fitting R2: 0.865
Novel peptide residual Z score: −0.552
Number of peptides: 3253
Pred. hydrophobicity
80
60
40
20
0
0
20000
40000
60000
Experimental scan number
C
Distributions of residuals from best−fit line
of predicted RT vs Expt. scan number
Line: Z score of novel peptide
Z: −0.552
count
750
500
250
0
−10
−5
0
5
10
Z scores of residuals from best linear fit
